# Effect of nasogastric tube feeding on oral wound healing and oral health-related quality of life (OHRQoL) after surgery for medication-related osteonecrosis of the jaw (MRONJ)

**DOI:** 10.1186/s13005-025-00543-4

**Published:** 2025-10-03

**Authors:** Luise Surmann, Julian Lommen, Valentin Kerkfeld, Max Wilkat, Rita Depprich, Henrik Holtmann, Majeed Rana, Norbert R. Kübler, Lara Schorn

**Affiliations:** 1 Department of Oral-, Maxillofacial and Facial Plastic Surgery, University Hospital Düsseldorf, Moorenstraße 5, Düsseldorf, Germany; 2 Department of Face Aesthetics, LVATE, Cecilienallee 11, Düsseldorf, Germany

**Keywords:** Medication-related osteonecrosis of the jaw, MRONJ, Nasogastric tube, NGT, Oral wound healing, Oral health impact profile 14, OHIP-G 14, OHIP 14, OHRQoL

## Abstract

**Background:**

In patients with medication-related osteonecrosis of the jaw (MRONJ), the use of nasogastric tubes (NGTs) after surgery is recommended to allow adequate nutrition without food interfering with oral wound healing. However, NGT therapy is often perceived as irritating and rejected by some patients. This study evaluates the effect of NGT feeding on oral wound healing and patients' oral health-related quality of life (OHRQoL) after surgical treatment of MRONJ.

**Methods:**

We assessed early wound healing in 68 patients after Surgery for MRONJ. Postoperative comparison was made between patients with NGT feeding and a matched control group receiving an oral clear liquid diet. At 14 days postoperatively, the healing of the surgical site was morphologically classified as “complete” or “incomplete”. The Early Healing Score (EHS) and the Inflammatory Proliferative Remodeling (IPR) Scale were examined on Days 1, 5 and 14 after surgery. The German version of the Oral Health Impact Profile-14 (OHIP-G 14) was used to assess OHRQoL.

**Results:**

No significant difference was observed in the rate of complete wound healing in patients receiving NGT feeding (61.1%) compared to patients receiving an oral clear liquid diet (62.5%) at 14 days postoperatively. The mean total EHS and the IPR Scale were not significantly different between patients on NGT feeding (EHS: 18.08 ± 5.35, IPR Scale: 14.36 ± 3.08) and patients on an oral clear liquid diet (EHS: 18.03 ± 5.26, IPR Scale: 14.66 ± 3.24). Furthermore, there was no significant difference in the mean OHIP-G 14 total score regardless of NGT therapy or consumption of an oral clear liquid diet.

**Conclusions:**

The results indicate that postoperative NGT feeding has no beneficial effect on wound healing after surgical treatment of MRONJ. It can be assumed that NGT feeding can be replaced by an oral clear liquid diet after surgery for MRONJ without compromising oral wound healing. Additionally, our data suggest that there is no difference in OHRQoL between patients treated with an NGT and those treated with an oral clear liquid diet. Therefore, patients' negative perceptions of NGTs do not appear to affect OHRQoL.

Trial registration.

The trial was retrospectively registered with the German Clinical Trials Register on February 26, 2024 (DRKS00033706).

## Background

The medication-related osteonecrosis of the jaw (MRONJ) is a serious complication of antiresorptive or antiangiogenic drugs and poses an increasing challenge for Oral and Maxillofacial Surgery (OMS). The most commonly associated drugs with MRONJ are Bisphosphonates (BPs) and Denosumab (DNO) [1]. Recent data show that there is a growing list of drugs with antiresorptive properties e.g., angiogenesis inhibitors, tyrosine kinase inhibitors, immunomodulators and estrogen inhibitors [2, 3]. The application range of antiresorptive agents (ARAs) comprises the treatment and prevention of bone metastases from solid tumors and the therapy of primary bone tumors. Furthermore, ARAs are used in treating osteoporosis, hypercalcemia and skeletal dysplasia [3].

MRONJ is a rare adverse event after therapy with ARAs [4–6]. It is defined by the following criteria: history of antiresorptive therapy, exposed bone in the maxillofacial region for 8 weeks and the absence of radiation therapy in the maxilla and mandibula [7].

Treatment styles for manifest MRONJ range from conservative to surgical approaches for all stages of the disease. Conservative treatment alone is preferably used in initial stages and when comorbidities forbid surgical intervention [7]. The overall aim is to control the patients’ symptoms by reducing pain, preventing secondary infection and limit the spread of the disease [8, 9]. Conservative procedures usually involve antimicrobial oral rinses, removal of mobile sequestrum, systemic antibiotics and pain management [7, 9]. Although non-surgical therapy remains a treatment option, previous work has shown that surgical therapy is associated with a beneficial outcome at all stages [10, 11]. Surgical treatment is based upon surgical removal of necrotic bone until signs of bone bleeding appear. This is followed by smoothing remaining sharp bone edges [12, 13]. Establishing a full-thickness mucoperiosteal flap closure for tension-free wound-seal is crucial for postsurgical wound healing in patients with MRONJ [14, 15]. Systemic antibiotics before and after surgery offer additional benefits [16, 17]. Although several authors recommend long-term antibiotic therapy depending on the size and clinical situation of the lesion, there is no general agreement on the duration of treatment [18].

Due to the good blood circulation in the oral cavity, wounds in the mouth can heal within two weeks [19]. Intraoral wound healing is influenced by local and systemic factors. Unlike surgery in other parts of the body, wounds in the oral cavity are exposed to regular food intake. Therefore, postoperative dietary recommendations and restrictions are given to prevent mechanical, chemical, and thermal irritation of early wound healing [19, 20]. Oral supplementation, enteral feeding, or parenteral feeding may be used as further nutritional interventions. Some preliminary work has defined a stepwise approach to postoperative dietary guidelines consisting of clear liquids, full liquids, pureed foods, soft foods, and regular foods [19, 21, 22]. However, postoperative dietary recommendations are not standardized and vary considerably both nationally and internationally. Recommendations are often based on individual personal expertise and clinical routine [23].

In addition, in patients undergoing dentoalveolar surgery, nutrition plays an important role in wound healing. Nutritional deficiencies in carbohydrates, proteins, fat, vitamins, or minerals compromise oral tissue repair and bone healing [21]. Cancer patients and older people with osteoporosis, such as those with MRONJ, are particularly at risk of malnutrition and cachexia [24]. Surgical procedures in the oral cavity may further impair sufficient nutrition intake prior to and after dentoalveolar surgery [21, 24].

The guideline by the Association of the Scientific Medical Societies in Germany (AWMF, *Arbeitsgemeinschaft der Wissenschaftlichen Medizinischen Fachgesellschaften*) for MRONJ states that enteral nutrition via nasogastric tubes (NGTs) may temporarily replace oral nutrition [22, 25]. NGT feeding ensures the patient’s energy requirements and at the same time protects the mucosal seal and sutures from food irritation [26]. Despite their beneficial effects, NGTs are associated with tube-related, gastrointestinal and pulmonary complications [27]. Previous work showed that NGTs are poorly tolerated in some patients. Foreign body sensation in the throat, reflux esophagitis and dislocation are only some limitations of NGT feeding [27]. The psychological burden of prolonged NGT feeding, combined with restricted mobility and altered appearance, further affects patients' quality of Life (QoL) [27–29].

Health-related QoL (HRQoL) has become an important factor in medical research and decision-making [30]. In 1995 the World Health Organization defined QoL as "an individual's perception of their position in life in the context of the culture and value systems in which they live and in relation to their goals, expectations, standards and concerns" [31]. Since then, much research has been done in the field and the definition has gained complexity [32]. Oral HRQoL (OHRQoL) is a concept for assessing the impact of oral conditions on QoL [33]. The Oral Health Impact Profile (OHIP) 49 is a questionnaire that addresses the social impact of oral disease and is an established tool for assessing OHRQoL. OHIP 14 is an abbreviated version with 14 questions and is an effective replacement for OHIP 49 in clinical practice [34, 35].

Several OMS surgeons advocate postsurgical enteral nutrition via NGTs in patients with MRONJ. The AWMF MRONJ guideline mentions NGTs as a possible adjunct postoperative measure [22]. As far as we know, it has not yet been established whether NGT feeding improves wound healing in patients with MRONJ. Furthermore, to our knowledge, no one has studied the effect of NGTs on OHRQoL in patients with MRONJ.

In this study, we investigated whether NGT feeding leads to improved wound healing in patients with MRONJ after surgery. To answer this question, we investigated early wound healing in patients with NGT feeding after surgery compared with patients who received an oral clear liquid diet. Wound healing was assessed by clinical categorization as “complete” or “incomplete” and the use of oral wound scores. To test the hypothesis that NGTs cause a decrease in OHRQoL, we used the OHIP-G 14 questionnaire.

## Materials and methods

### Study participants

**Fig. 1:**
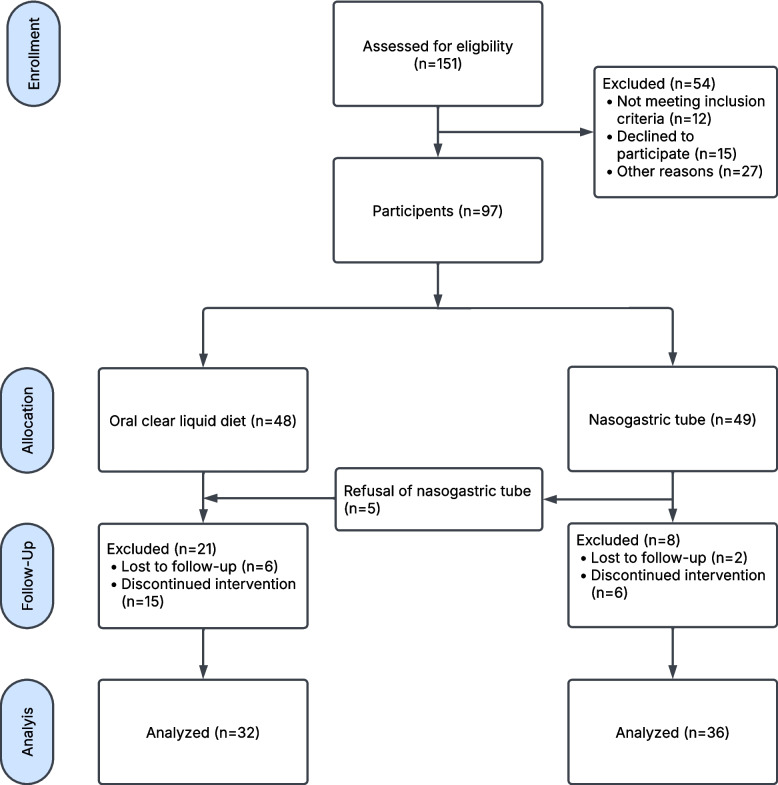
Flow chart of the study

68 MRONJ patients undergoing surgery at the Department of Oral and Maxillofacial Surgery at Heinrich Heine University, Düsseldorf, Germany, between 2020 and 2023 were included in the trial. Inclusion criteria was a diagnosis of MRONJ as defined by the American Association of Oral and Maxillofacial Surgeons (AAOMS) [8]. MRONJ patients with an entirely extraoral surgical approach and patients with insufficient German language skills were excluded from the study (Fig. 1).

### Study design

A prospective controlled trial was conducted. Patients were admitted to hospital the day before surgery for preoperative antibiotic treatment. Antimicrobial therapy consisted of the penicillin beta-lactamase inhibitor combination ampicillin/sulbactam 2 g/1 g 1–1-1 intravenous (Pfizer Pharma GmbH, Berlin, Germany) in hospital and amoxicillin/clavulanic acid 875 mg/125 mg 1–1-1 per os after discharge. In case of penicillin allergy, the regime was Substituted by clindamycin 600 mg 1–1-1 intravenous and per os. Antibiotic therapy was continued until clinical signs of surgery and bacterial contamination had subsided.

**Fig. 2:**
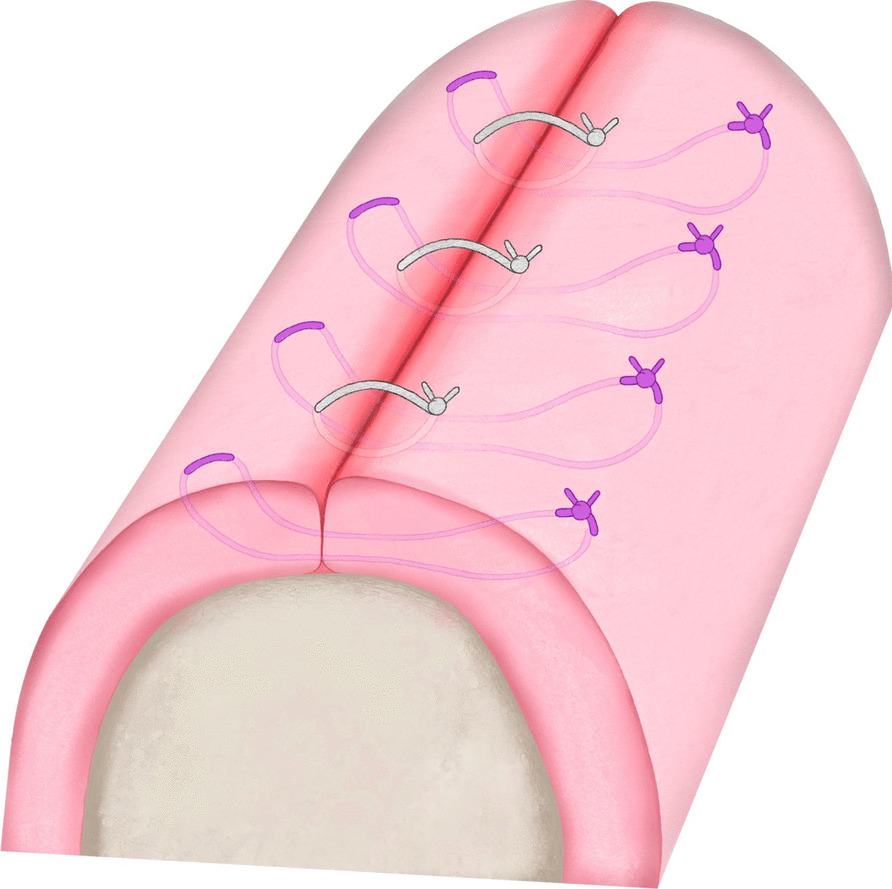
Graphic illustration of double layered saliva-proof mucosal seal with Vicryl® 3–0 (purple sutures) and Supramid® 3–0 (grey sutures)

Surgical procedures were performed with local or general anesthesia, depending on the extent of the lesion and the patient’s general condition. Tooth extraction and implant removal were performed when necessary. Bone that was macroscopically involved was resected until fresh bone bleeding was observed. Any sharp bone edges were smoothed using rotary instruments. Depending on the incision and the extent of the defect, local flap Surgery and a periosteal release incision were chosen for tension-free adaptation of the wound edges. A saliva-proof wound closure was performed in two layers. Horizontal mattress Sutures were placed in depth with Vicryl® 3–0 (Johnson & Johnson Medical GmbH/ETHICON, Norderstedt, Germany), but were not yet knotted. This was followed by adaptation of the wound edges with simple interrupted Sutures using Supramid® 3–0 (RESORBA Medical GmbH, Nürnberg, Germany). Finally, the horizontal mattress sutures were knotted (Fig. 2). Tissue samples taken during the operation were subjected to histopathological examination to rule out a malignant event and to confirm the diagnosis of MRONJ. For postoperative management, an antibacterial mouth rinse with Chlorhexamed® FORTE 0,2% (GlaxoSmithKline Consumer Healthcare GmbH & Co. KG, Munich, Germany) was used in all patients for 14 days.

Patients were assigned to two groups using a dichotomous randomization method. The first patient received treatment with an NGT, the second did not, and subsequent patients alternated between NGT treatment and an oral clear liquid diet. If a patient declined NGT insertion prior to the operation, they were reassigned to the group receiving the oral clear liquid diet. In the test group the NGT (Silicone Dual Lumen Stomach Tube 16 Fr/Ch, Covidien, Medtronic, Dublin, Ireland) was inserted after Surgery and removed when the patient was discharged 5 days after surgery. In cases of advanced disease or delayed wound healing, treatment with an NGT was continued for a longer period. Deviations in the number of days due to compliance also occurred in few cases. Patients received tube feeds (Fresubin®, Fresenius Kabi AG, Bad Homburg, Germany) and boiled water according to their nutritional needs. In the control group, patients were given a clear liquid diet consisting of water, unsweetened tea, clear soup and a high caloric energy drink (ProvideXtra® Drink 200 ml, Fresenius Kabi AG, Bad Homburg, Germany) for 5 days. Afterwards, all patients were advised to follow a staged oral diet, starting with full liquids, followed by pureed and soft foods.

To investigate the efficacy of NGT nutrition, clinical wound healing criteria were assessed. The Early Healing Score (EHS) [36] and the Inflammatory Proliferative Remodeling (IPR) Scale [37] were modified to monitor postoperative oral mucosal wound healing following MRONJ surgery. The OHIP-G 14 questionnaire was used to determine the impact on OHRQoL.

The study was conducted in accordance with the Declaration of Helsinki and approved by the Ethics Committee of the Medical Faculty of Heinrich-Heine-University Düsseldorf (reference: 2018–244-KFogU). After a precise explanation of the procedure, written informed consent was obtained from each patient who agreed to participate in the study.

### Procedures

The clinical examinations were performed 1 day before and 1, 5 and 14 days after surgery by a single qualified practitioner who was not the operating surgeon.

At the preoperative visit, patients' medical histories were taken and the MRONJ staging, according to the AAOMS, was performed. Photo documentation of MRONJ lesions with an intraoral camara (EyeSpecial C-III, SHOFU Dental GmbH, Ratingen, Germany) was taken. Additionally, the OHIP-G 14 questionnaire was given.

During Postoperative visits, participants underwent an oral examination to assess wound healing. The primary outcome was the final appearance of the wound at 14 days postoperatively. Healing outcomes were classified as “complete” or “incomplete”. The surgical site was clinically evaluated by morphological means. Complete wound healing was defined as maintenance of the seal of the oral mucosa without any symptoms. Incomplete wound healing was defined by the dehiscence of the wound margins, the presence of exposed bone, purulent drainage, intraoral or extraoral fistula, or pain associated with the surgical site. Wound healing scales were used to monitor the development of oral tissue repair after surgery over time. Wound scoring was performed using two independent indices described in recent OMS literature, the EHS and IPR Scale [36, 38].

The EHS is a validated tool for assessing early postoperative wound healing of intraoral soft tissues [36]. The index evaluates clinical signs of re-epithelialisation (CSR), haemostasis (CSH) and inflammation (CSI). CSR is scored with 0, 3 or 6 points, while CSH and CSI are scored with 0, 1, or 2 points. The Sum of the 3 scores equals the EHS. The total score of the EHS ranges from 0 to 10 points. The higher the EHS, the better the wound healing. The total scores of the 3 parameters were recorded at the examination on day 1, 5 and 14 after surgery.

The IPR Scale monitors oral soft tissue repair corresponding to the 3 phases of wound healing [37, 38]. The inflammatory, proliferative and remodeling phase are weighted in descending order in the healing process. 8 clinical parameters are rated in the inflammatory phase, 5 in the proliferative phase and 3 in the remodeling phase. Each parameter is scored 0 or 1. The higher the IPR Scale, the better the wound healing. During follow-up, oral mucosal wound healing was evaluated using the appropriate Subscale. The inflammatory phase Subscale was assessed on Days 1 and 5, whereas the proliferative phase Subscale was assessed on day 14. The remodeling phase was not evaluated in this study.

OHRQoL was assessed 1 day before and 1 and 5 days after surgery using OHIP-G 14, the German version of OHIP 14. Additionally, QoL was measured by asking patients how much they currently felt their QoL was affected, using a visual analogue scale (VAS) from 1 (not at all) to 10 (extremely).

### Statistics

Data were analyzed using SPSS Statistics Version 26.0 (IBM SPSS Statistics for Macintosh, Version 26.0. Armonk, NY: IBM Corp). Categorical variables were compared using Pearson's chi-square test. The Shapiro–Wilk test was used to examine normality in the distribution of the continuous variables. Comparisons were made using the unpaired Student t-test. Repeated measures ANOVA was used to assess the differences in the EHS, the IPR Scale and the OHIP-G 14 between the groups and at different time points. To investigate whether the duration of NGT feeding influenced wound healing, binomial logistic regression was performed with wound healing as the dependent variable and days of NGT feeding as the independent variable. In addition, to account for the influence of potential confounding variables, we performed binary logistic regression, ANCOVA and repeated measures ANCOVA. Data were expressed as mean ± SD. The statistical significance level was set at *p* ≤ 0.05. Graphs were generated using GraphPad Prism version 10.1.1 for MacOS, GraphPad Software, Boston, Massachusetts USA.

## Results

### Characteristics of study population

First, we compared patient characteristics of the experimental and the control group to provide detailed information about the study population and to look for significant differences in patient characteristics that might affect the interpretation of the study results and introduce bias. The only significant differences between the groups were found for body mass index (*p* = 0.005), the site of the operation being in the lower jaw (*p* = 0.024), and the performance of a sequestrectomy (*p* = 0.041). We conducted statistical tests to determine whether the variables "lower jaw", "sequestrectomy", and "body mass index" had any unintended influence on our results regarding wound healing. In the first analysis, a binary logistic regression was performed, with wound healing (complete/incomplete wound healing) as the dependent variable and the type of diet (NGT vs. oral clear liquid diet) as the independent variable. The results indicated that the covariates "lower jaw" (*p* = 0.206), "sequestrectomy" (*p* = 0.092), and "body mass index" (*p* = 0.139) did not have a significant impact on wound healing. Next, an ANCOVA was conducted, using the mean total EHS and mean total IPR Scale as the dependent variables. Similar to the previous analysis, the covariates "lower jaw" (mean total EHS: *p* = 0.990; mean total IPR Scale: *p* = 0.879), "sequestrectomy" (mean total EHS: *p* = 0.062; mean total IPR Scale: *p* = 0.480), and "body mass index" (mean total EHS: *p* = 0.878; mean total IPR Scale: *p* = 0.590) did not significantly influence the wound healing outcomes. Finally, a repeated measures ANCOVA was performed, with mean EHS and mean IPR Scale as the dependent variables. The findings from this analysis were consistent with the previous results, showing no significant effect of the covariates "lower jaw" (mean EHS: *p* = 0.814; mean IPR Scale: *p* = 0.524), "sequestrectomy" (mean EHS: *p* = 0.554; mean IPR Scale: *p* = 0.676), or "body mass index" (mean EHS: *p* = 0.243; mean IPR Scale: *p* = 0.448) on wound healing. In summary, after adjusting for the covariates "lower jaw," "sequestrectomy," and "body mass index," no statistically significant difference in wound healing was found, regardless of the type of diet. Other patient characteristics were similar at baseline and throughout the study (Table 1). Overall, our treatment group was comparable to our control group in patient characteristics.

**Table 1:** Characteristics of study population

	NGT (*n* = 36)	Oral clear liquid diet (*n* = 32)	*p* Value
Gender, n			
Female	24 (66.7%)	20 (62.5%)	0.720
Male	12 (33.3%)	12 (37.5%)	
Age, mean (range) yr	68.69 (43–85)	65.00 (35–85)	0.242
Health insurance, n			
Statutory	23 (63.9%)	27 (84.4%)	0.056
Private	13 (36.1%)	5 (15.6%)	
Reason for antiresorptive therapy, n			
Osteoporosis	2 (5.6%)	4 (12.5%)	0.314
Malignant disease	34 (94.4%)	28 (87.5%)	
ASA, n			
I	5 (14.3%)	2 (6.5%)	0.549
II	10 (28.6%)	11 (35.5%)	
III	20 (57.1%)	18 (58.1%)	
IV	0 (0%)	0 (0%)	
Type of antiresorptive medication, n			
Zolendronic acid	7 (21.2%)	7 (23.3%)	0.931
Densoumab	20 (60.6%)	16 (53.3%)	
Zolendronic acid and Denosumab	3 (9.1%)	3 (10.0%)	
Other	3 (9.1%)	4 (13.3%)	
Duration of antiresorptive therapy, mean (range) mo	51.15 (5–183)	38.61 (6–120)	0.173
Risk group, n			
1	2 (5.6%)	5 (16.1%)	0.252
2	1 (2.8%)	0 (0%)	
3	33 (91.7%)	26 (83.9%)	
Diabetes mellitus	5 (13.9%)	4 (12.5%)	0.866
Medication, n			
Blood thinners	14 (38.9%)	12 (37.5%)	0.906
Immunosppressants	9 (25.0%)	10 (31.3%)	0.566
Hormone therapy	15 (41.7%)	15 (46.9%)	0.666
Chemotherapy	13 (36.1%)	6 (18.8%)	0.111
Body mass index, mean (range)	25.78 (17.53–37.18)	22.76 (17.53- 33.26)	*0.005
Smoking, n	6 (16.7%)	9 (29%) (*n* = 31)	0.226
Alcohol, n	16 (47.1%)	17 (56.7%) (*n* = 30)	0.443
Site, n			
Upper jaw	6 (16.7%)	11 (34.4%)	0.092
Lower jaw	34 (94.4%)	24 (75.0%)	*0.024
AAOMS stage, n			
0	0 (0%)	3 (9.4%)	0.234
1	21 (58.3%)	15 (46.9%)	
2	14 (38.9%)	12 (37.5%)	
3	1 (2.8%)	2 (6.3%)	
Length of hospital stay after the operation, mean (range) d	6.81 (3–20)	6.03 (3–26)	0.375
Surgery, n			
Implant removal	1 (2.8%)	2 (6.3%)	0.486
Tooth removal	16 (44.4%)	12 (35.5%)	0.561
Tooth root removal	0 (0%)	2 (6.3%)	0.128
Debridement	36 (100%)	30 (93.8%)	0.128
Sequestrectomy	15 (41.7%)	6 (18.8%)	*0.041
Block resection	0 (0%)	0 (0%)	n.a
Continuity resection	1 (2.8%)	1 (3.1%)	0.933
Antibiotics, n			
Ampicillin/sulbactam and amoxicillin/clavulanic acid	28 (77.8%)	19 (59.4%)	0.260
Clindamycin	6 (16.7%)	10 (31.3%)	
Other	2 (5.6%)	3 (9.4%)	
Length of antibiotic therapy, mean (range) d	15.50 (6–49)	15.88 (4–36)	0.846

*NGT* nasogastric tube, *ASA* American Society of Anesthesiologists, *AAOMS* American Association of Oral and Maxillofacial Surgeons ^*^, *p* < 0.05

### Oral wound healing

#### Complete/incomplete wound healing

First, we analyzed the rate of complete and incomplete wound healing by clinical assessment at 14 days Postoperatively in the total study population. 68 patients were studied. Complete wound healing was observed in 42 patients (61.8%). Incomplete wound healing was observed in 26 patients (38.2%), regardless of diet.

We then asked whether NGT feeding improves the rate of complete wound healing after Surgical treatment of MRONJ, as determined by clinical assessment of the Surgical site at 14 days Postoperatively. A total of 68 patients were examined. Of these, 36 patients were fed via NGT, and 32 patients received an oral clear liquid diet. In patients with NGT feeding complete healing of the Surgical site was observed in 22 patients (61.1%). Correspondingly, incomplete healing was found in 14 patients (38.9%). In patients on an oral clear liquid diet, complete healing of the Surgical site was observed in 20 patients (62.5%). Incomplete healing was observed in 12 patients (37.5%). There was no statistically significant association between diet type and wound healing outcome (*p* = 0.91). In conclusion, NGT feeding did not improve complete wound healing rates after surgical treatment of MRONJ.

#### Mean total EHS

The mean total EHS of Postoperative day 1, 5 and 14 was compared between the patients who received NGT feeding and the patients who received an oral clear liquid diet. The Sum of the three points in time was calculated to compare wound healing over the entire period. There were 36 participants in the experimental group and 32 participants in the control group (*n* = 68). The mean sum in patients with NGT feeding (18.08 ± 5.35) was only 0.05 points higher than in the comparison group (18.03 ± 5.26). The difference between the groups was not statistically significant (*p* = 0.97). In conclusion, the EHS showed no difference in the mean total number of the three examination times in patients with an NGT compared with patients on an oral clear liquid diet.

#### Mean EHS

**Fig. 3:**
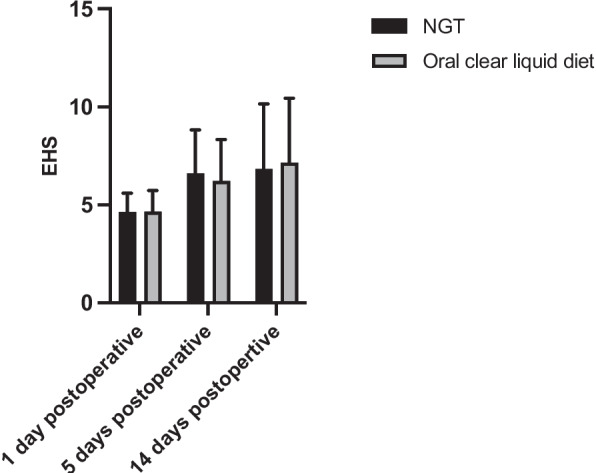
Early Healing Score (EHS) in patients with nasogastric tube (NGT) feeding and patients with an oral clear liquid diet 1, 5 and 14 days postoperatively. Results were presented as mean ± SD. (1 day postoperative: NGT = 4.64 ± 0.96, oral clear liquid diet = 4.66 ± 1.07; 5 days postoperative: NGT = 6.61 ± 2.21, oral clear liquid diet = 6.22 ± 2.11; 14 days postoperative: NGT = 6.83 ± 3.33, oral clear liquid diet = 7.16 ± 3.29;* p* = 0.52)

Individual assessments of the EHS at 1, 5 and 14 days postoperatively were performed to analyze whether NGT feeding improved early wound healing after Surgery for MRONJ lesions at different time points in the healing process. The mean EHS showed a steady increase over time on Days 1, 5 and 14 after surgery in both the experimental and control group (Fig. 3). The results showed that the mean EHS increased, statistically, over time with a significant increase from day 1 to day 5 (*p* < 0.001) and from day 1 to day 14 (*p* < 0.001). The mean EHS was not significantly different between the experimental and control group (*p* = 0.52). Overall, NGT feeding after Surgery for MRONJ lesions did not increase the mean EHS at 1, 5 and 14 days, postoperatively, in comparison to an oral clear liquid diet.

#### Mean total IPR scale

The mean total IPR Scale of Postoperative day 1, 5 and 14 was compared between the patients who received NGT feeding and the patients who received an oral clear liquid diet. This was done to compare wound healing over the entire period. There were 36 participants in the experimental group and 32 participants in the control group (*n* = 68). The mean sum in patients with NGT feeding (14.36 ± 3.08) was only 0.3 points lower than in the comparison group with an oral clear liquid diet (14.66 ± 3.24). The difference between the groups was not statistically significant (*p* = 0.70). In summary, the IPR Scale showed 

#### Mean IPR scale

**Fig. 4:**
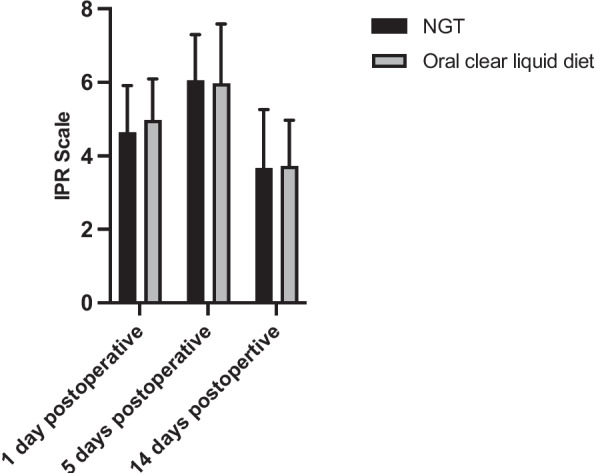
Inflammatory Proliferative Remodeling (IPR) scale 1, 5 and 14 days postoperatively in patients with nasogastric tube (NGT) feeding and patients with an oral clear liquid diet. Results were presented as mean ± SD. (1 day postoperative: NGT = 4.64 ± 1.27, oral clear liquid diet = 4.97 ± 1.12; 5 days postoperative: NGT = 6.06 ± 1.24, oral clear liquid diet = 5.97 ± 1.62; 14 days postoperative: NGT = 3.67 ± 1.59, oral clear liquid diet = 3.72 ± 1.25; *p* = 0.51)

Individual Assessment of the mean IPR Scale at 1, 5 and 14 days after surgery was performed to analyze whether NGT feeding improves early wound healing after surgery for MRONJ lesions at different points in time during the healing process. A general assessment of wound healing over time is not meaningful with the IPR Scale, as the maximum score that can be achieved at the individual points in time is different (8 points: Postoperative day 1 and 5, 5 points: Postoperative day 14). When comparing the groups, the mean IPR Scale was not significantly different between the experimental and control group at any point in time (*p* = 0.51) (Fig. 4). In none of the three study points did the use of an NGT lead to a change in the mean IPR Scale compared to an oral clear liquid diet.

#### Complete/incomplete wound healing and duration of NGT feeding

We investigated whether prolonged NGT feeding would improve complete wound healing. The clinical outcome of wound healing 14 days after Surgery and the mean number of Days of NGT feeding were considered. 36 patients received NGT feeding. Of those, 22 patients showed complete wound healing. The mean duration of NGT feeding was approximately 6 days (5.50 ± 1.44). There were 14 patients with incomplete wound healing and the mean duration of NGT feeding was also approximately 6 days (6.29 ± 2.52). A binomial logistic regression was performed with wound healing (complete/incomplete wound healing) as the dependent variable and duration of NGT treatment (days of NGT) as the independent variable. The number of days of NGT feeding showed no significant influence (*p* = 0.24) on the outcome of wound healing at 14 days Postoperatively with an odds ratio of 0.80. It can be concluded, that prolonged NGT feeding did not improve complete wound healing.

### Oral health-related quality of life

#### OHIP-G 14 preoperatively

Focusing on QoL, we first analyzed preoperative OHRQoL in the entire study population by assessing OHIP-G 14. We also assessed OHIP-G 14 preoperatively according to the use of dentures, as recommended in the literature. The mean OHIP-G 14 total score in patients with MRONJ one Day before the operation was 14.59 ± 11.41. According to the use of dentures, the mean OHIP-G 14 total score was 13.73 ± 10.84 without removable dentures, 15.30 ± 11.66 with removable dentures, and 16.17 ± 14.85 with complete dentures.

#### OHIP-G 14

**Fig. 5:**
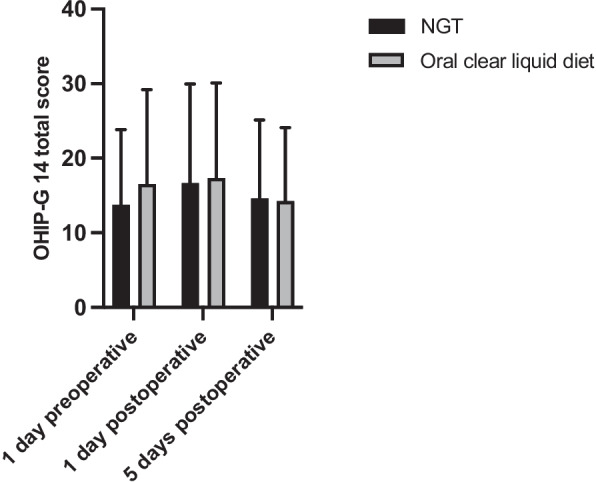
Oral Health Impact Profile-G 14 (OHIP-G 14) total score in patients receiving nasogastric tube (NGT) feeding and patients receiving an oral clear liquid diet 1 day preoperatively and 1 and 5 days postoperatively. Results were presented as mean ± SD. (1 day preoperative: NGT = 13.76 ± 10.06, oral clear liquid diet = 16.52 ± 12.67; 1 day postoperative: NGT = 16.68 ± 13.27, oral clear liquid diet = 17.30 ± 12.77; 5 days postoperative: NGT = 14.60 ± 10.56, oral clear liquid diet = 14.26 ± 9.83; *p* = 0.55)

We then asked whether NGT feeding had any effect on OHRQoL, as assessed by the mean OHIP-G 14 total score 1 day before Surgery and 1 and 5 days after surgery. Results showed no significant change in the mean OHIP-G 14 total score at the recorded time points (*p* = 0.16) (Fig. 5). Additionally, there was no significant difference in the mean OHIP-G 14 total score between patients on NGT therapy and patients on an oral clear liquid diet (*p* = 0.55). As a result, there was no significant difference in OHIP-G 14 between NGT feeding and oral clear liquid diet over time and at any of the time points recorded.

#### Impact on QoL

We then assessed patients' QoL on Postoperative Days 1 and 5 by asking how much they felt their QoL was currently affected, using a VAS ranging from 1 (not at all) to 10 (extremely). On Postoperative day 1, there were 30 participants in the experimental group and 24 participants in the control group (*n* = 54). According to the VAS, the mean limitation of QoL in patients with NGT (6.10 ± 3.07) was 0.1 points higher than in the comparison group (6.00 ± 2.92). The difference between the groups was not statistically significant (*p* = 0.90). On Postoperative day 5, there were 27 participants in the experimental group and 24 participants in the control group (*n* = 51). According to the VAS, the mean limitation of QoL in patients with an NGT (5.44 ± 2.49) was 0.1 points lower than in the comparison group (5.54 ± 2.69). The difference between the groups was not statistically significant (*p* = 0.89). Consequently, the use of NGTs was not associated with a reduction in QoL as measured by a VAS at 1 and 5 days postoperatively.

## Discussion

In this study, we found that postoperative NGT feeding has no beneficial effect on wound healing after Surgical treatment of MRONJ. 14 days after the operation, there was no significant difference in complete wound healing between patients on NGT feeding and those on an oral clear liquid diet. Similarly, oral wound healing scores did not differ between the groups. Additionally, our results indicate that NGT feeding does not affect OHRQoL. On Postoperative day 5, there was no significant difference in OHIP-G 14 scores between patients with NGT feeding and those on an oral clear liquid diet.

The AWMF MRONJ guideline suggests that optional perioperative supportive measures include a gradual change in diet or bypassing oral intake with NGT feeding [22]. To date, there is no reliable data on the benefits of these measures in patients with MRONJ. Experiments on the effect of soft foods on human gingival epithelial cell growth were conducted in 2017 by a group of researchers from Canada. They investigated the interaction between a choice of soft foods (orange juice, drinkable yogurt, nutritional drink) and human gingival epithelial cells in vitro. The soft food did not have an adverse effect on gingival epithelial cell proliferation. Conversely, there was evidence of a beneficial effect through stimulation of epithelial cells via keratin expression and cytokine release [39]. These findings Suggest that neither NGT feeding, nor a clear liquid diet would improve oral wound healing while an earlier start to oral nutrition may be beneficial. Due to the lack of clinical data, these results should be interpreted with utmost caution. A recent study by Moghaddam et al. on this Subject revealed that, particularly in German and Flemish speaking countries, more than 50% of the dental and maxillofacial surgeons support the view that dairy products should be avoided after oral surgery [23]. While in other countries there is no such recommendation, in countries such as the USA, Italy, Thailand and India, practitioners do strongly recommend the consumption of milk and dairy products. They found that there was no evidence to support the harmful effects of milk and dairy products [23]. Our data show some similarity in that bypassing oral food intake with an NGT did not improve oral wound healing. However, the results should be interpreted with care as patients in our study were only allowed to drink clear liquids for 5 days. Fahim et al. showed that oral wound healing in rats was impaired by the consumption of carbonated beverages. They hypothesized that the hyperplasia and hyperkeratosis of the oral epithelium was caused by irritation from the acids and carbonation of drinks [40]. Contrary to the above, these results support a strict adherence to diet in the postoperative period after oral surgery, especially one without the consumption of carbonated beverages.

Intraoral wound healing is especially impaired in patients with MRONJ. The pathology of MRONJ and its specificity to the jaw is still poorly understood. Suppressed bone remodeling, inflammation and inhibited angiogenesis are central to the pathogenesis of MRONJ. As a result, altered wound healing of the bone and soft tissues of the maxillofacial region is a difficult clinical challenge. A recent review of the literature on the effect of BPs on the oral mucosa in MRONJ found that BP treatment is toxic to oral soft tissue. The studies reviewed show that BP therapy reduces cell viability and proliferation while increasing apoptosis in oral keratinocytes and fibroblasts [41]. For this reason, wound healing in patients following resection of MRONJ lesions and intraoral soft tissue closure is particularly vulnerable. This is reflected in a high rate of recurrence with dehiscence of the intraoral mucosa after completion of MRONJ therapy. In the present study the Success rate for Surgical treatment of BRONJ was 61.8%. This is consistent with the literature where cure rates for MRONJ after Surgery range between 55 and 89% [14, 42, 43].

Patients with MRONJ often have nutritional deficiencies for three reasons. Firstly, many cancer and osteoporosis patients are malnourished. Secondly, oral surgery and MRONJ make it difficult to chew and swallow, increasing the risk of malnutrition [21, 24, 44, 45]. Thirdly, In case of surgery and disease the metabolic demand is increased and even more energy is needed [21]. It is therefore not Surprising that 56% of those affected by MRONJ were identified to be at risk of becoming malnourished [24]. An increasing number of studies have shown that poor nutrition is associated with a delay in wound healing and immune system dysfunction with increased susceptibility to infection [21, 24, 46]. Postoperative restrictions on oral intake can further complicate nutritional management. Although the patients in the control group were allowed to consume clear energy drinks, there was concern that their nutrient intake was inferior to that of the NGT diet. Interestingly there was no impaired wound healing in patients with a clear liquid diet compared to NGT treatment. Therefore, it can be assumed that there is no significant difference in nutrition that affects wound healing when comparing NGT feeding with an oral clear liquid diet.

NGT feeding can maintain nutritional therapy when the patient's ability to eat is impaired [44]. In addition to NGT therapy, there are several ways to provide enteral nutrition. In OMS, NGTs and percutaneous endoscopic gastrostomy (PEG) are the most commonly used. NGTs are indicated for short-term use of 4–6 weeks. Long-term enteral nutrition, which is often required in patients with head and neck cancer, is mainly provided via PEG [27]. The European Society for Clinical Nutrition and Metabolism (ESPEN) recommends tube feeding within the first day after surgery if oral intake is impossible or insufficient for a week [47]. The benefits of NGT therapy are numerous. The use of NGTs minimizes contamination of the wound with nutrients for pathogens. NGTs also prevent mechanical trauma to the wound from the food intake and chewing. In addition, a recent study found that the use of an NGT for 5 days after surgery in patients with MRONJ resulted in a stable nutritional status of the parameters assessed [24]. Further, NGT therapy is superior to parenteral nutrition in terms of outcomes, complications, and cost [27]. Evidence suggests that NGT therapy improves wound healing, shortens hospital stays and increases life expectancy [28]. However, NGT feeding is also known to cause complications. Gastrointestinal, metabolic, or mechanical tube-related complications occur in 10% of patients [27, 44]. There are no standards for checking the position of NGTs, which are primarily inserted blindly. Preliminary studies have shown that 0.3—15.9% of the patients experience a misplacement in the trachea or lungs, a potentially life-threatening condition [48–50]. Another serious complication of NGT therapy is aspiration pneumonia [51]. Further investigations demonstrated an altered composition of the oral microbiome associated with long-term NGT feeding [51]. The analysis of the oral cavity revealed an increase in opportunistic pathogens such as Pseudomonas and Corynebacterium, which can cause aspiration pneumonia [51]. Contrary to expectations, we did not find any improvement in the healing of the oral wounds in the patients who were fed with an NGT after surgery for MRONJ. Our study was small, but given the serious complications, the benefits of NGTs in patients with MRONJ should be further investigated.

Although the management of patients with MRONJ has become a critical issue in research, a major drawback is the lack of standardized instruments to assess wound healing [52]. In line with previous literature, we assessed oral wound healing in terms of complete and incomplete wound healing [53]. We chose to assess wound healing on the Day the stitches were removed, 14 days after surgery, which is the time it takes for oral wounds to heal [19]. Some authors argue that the follow-up period for the evaluation of the resolution of MRONJ should be at least six months [52]. We decided on a shorter endpoint because we wanted to best assess the effect of NGT feeding on early wound healing, minimize patient burden, and make the trial practical.

Current literature discusses different scores for the assessment of intraoral wounds. To monitor the wounds over time, we decided to use two independent scoring instruments as there is no consensus on one scale and each emphasizes different parameters. It was decided that the best method to monitor the early phase of wound healing was the EHS, introduced by Marini et al. in 2018 [36]. A modified version of the IPR Scale was chosen as a second instrument in order to distinguish among the different phases of wound healing [38]. The independent evaluation of the two instruments allowed comparable conclusions to be drawn about intraoral wound healing in our study.

Because of prejudice or previous experience, some patients refuse to have an NGT inserted. In some cases, NGT feeding that has already started must be stopped early because patients find the NGT uncomfortable. Based on these clinical observations, we included the role of NGT therapy on patients' OHRQoL in our study. We were surprised to find that NGTs did not affect OHRQoL in our patients.

Understanding QoL has become an important aspect of medical decision making in recent years [30]. The OHRQoL concept is widely accepted for measuring the impact of various oral health conditions [33]. The studies reviewed show that MRONJ significantly impairs OHRQoL [54–56]. The stage of disease correlates with the impairment of OHRQoL [54, 57]. Next to other instruments OHIP 14 was widely used to assess OHRQoL in patients with MRONJ. It is proven to be an effective tool for the assessment of OHRQoL [58].

Caminah et al. have established thresholds for OHIP 14 that define a weak (< 9.33), medium (9.33–18.66) or strong (> 18.66) impact on OHRQoL [58]. In the case of MRONJ, OHIP 14 scores range from 10.72–21.10 [56, 57, 59]. Our study population had a mean OHIP-G 14 score of 14.59, which agrees with previous literature findings. John et al. developed reference values for the interpretation of OHIP-G 14 [60]. The population-based norms of OHIP-G 14 were defined as 0 without removable dentures, 4 with removable dentures and 6 with complete dentures. Our mean OHIP-G 14 total scores of 13.73 without removable dentures, 15.30 with removable dentures, and 16.17 with complete dentures show a major impairment of OHRQoL in our patients, even before surgery. The data is consistent with previous data and emphasizes the need to focus on patients' QoL. Further deterioration in QoL due to additional medical interventions should be avoided at all costs.

To our knowledge, this is the first evaluation of OHRQoL in patients with MRONJ and NGT therapy. Data on QoL and NGT feeding in the recent literature is inconsistent. A systematic review of the effect of enteral tube feeding on HRQoL by Ojo et al. concluded that enteral tube feeding may be effective in improving HRQoL [28]. Unfortunately, the review includes different gastrostomy tubes and indications so that there is still uncertainty regarding NGTs and HRQoL. In case of digestive cancer, a program on therapeutic patient education with self-insertion training reached an overall NGT acceptability of nearly 80% with an improvement of QoL [61]. This provides evidence to support the hypothesis that better patient education may be a possible solution to improve patient acceptance of NGT feeding in clinical practice. In head and neck cancer patients, a randomized clinical trial showed a positive effect on QoL when NGT feeding was conducted in the period preceding surgery [62]. QoL in patients with acute pancreatitis treated with NGT versus nil-by-mouth was compared in a recent randomized trial. Given that they did not find that QoL worsened, they considered NGT therapy to represent a first-line approach [63]. Baker et al. investigated whether early enteral feeding improves QoL in patients with epithelial ovarian cancer. They concluded that early enteral feeding does not improve QoL compared with oral food intake but may improve nutritional status [64]. In a prospective study on QoL, the impact of home enteral tube feeding in patients with head and neck or oesophageal cancer was assessed. NGT feeding was used in 80% of the patients. They showed that the psychosocial wellbeing of the patients was compromised in terms of body image and social interaction while the physical feeding procedure was well accepted [65].

Unlike other research carried out in this area, we did not find that OHRQoL improved or worsened in patients with NGT feeding. When interpreting the results, it must be considered that a comparison was made with patients receiving a clear liquid diet. People on clear liquid diets are also likely to have a poor OHRQoL. It is also important to note that patients who refused to have an NGT inserted prior to surgery were assigned to the clear liquid diet control group. There is some likelihood that this may have influenced the OHRQoL results, as these patients may have had more severe impairment of OHRQoL. Our results suggest, contrary to our expectations, that the use of NGTs did not have a detrimental effect on the OHRQoL of our patients. Further studies are required to verify these findings.

This study has three major limitations. The first limitation lies in the fact that only a small number of patients was included in the study. Expanded data collection is required. The second limitation is the lack of a long-term evaluation. In this study, wound healing was only analyzed 14 days after the procedure. Therefore, changes in wound healing after this period were not considered. The third, and most important, limitation is a result of the fact that patients were allowed to refuse NGT therapy and were included in the control group. Consequently, the results on OHRQoL must be interpreted with care.

## Conclusions

The results suggest that postoperative feeding with NGTs has no beneficial effect on wound healing after surgical treatment of MRONJ compared to an oral clear liquid diet. It is assumed that postoperative NGT feeding can be replaced by an oral clear liquid diet after surgical therapy for MRONJ without compromising oral wound healing. Surprisingly, however, patients' OHRQoL was not affected by NGT feeding. Therefore, our results suggest that patients' concerns about NGTs don't affect OHRQoL. Within its limitations this study contributes to the development of a standardized diet for patients undergoing surgical treatment for MRONJ. Further studies analyzing wound healing are needed. To establish dietary guidelines, more work is needed on NGT feeding compared to full liquids and soft foods.

## Data Availability

No datasets were generated or analysed during the current study.

## Abbreviations

AAOMS: American Association of Oral and Maxillofacial Surgeons
ARA: Antiresorptive agent
ASA: American Society of Anesthesiologists
AWMF: *Arbeitsgemeinschaft der Wissenschaftlichen Medizinischen Fachgesellschaften, *Association of the Scientific Medical Societies in Germany
BP: Bisphosphonate
CSH: Clinical signs of haemostasis
CSI: Clinical signs of inflammation
CSR: Clinical signs of re-epithelialisation
DNO: Denosumab
EHS: Early Healing Score
HRQoL: Health-related quality of life
IPR Scale: Inflammatory Proliferative Remodeling Scale
MRONJ: Medication-related osteonecrosis of the jaw
NGT: Nasogastric tube
OHIP: Oral Health Impact Profile
OHRQoL: Oral health-related quality of life
QoL: Quality of life
VAS: Visual analogue scale
